# Use Patterns, Flavors, Brands, and Ingredients of Nonnicotine e-Cigarettes Among Adolescents, Young Adults, and Adults in the United States

**DOI:** 10.1001/jamanetworkopen.2022.16194

**Published:** 2022-05-25

**Authors:** Shivani Mathur Gaiha, Crystal Lin, Lauren Kass Lempert, Bonnie Halpern-Felsher

**Affiliations:** 1 Stanford Reach Lab, Division of Adolescent Medicine, Department of Pediatrics, Stanford University, Stanford, California

## Abstract

**Importance:**

Nonnicotine e-cigarettes contain chemicals, flavorants, and solvents that have known health harms and/or have not been proven safe for inhalation.

**Objective:**

To evaluate nonnicotine e-cigarette use patterns, including common flavors, brands, and ingredients.

**Design, Setting, and Participants:**

This cross-sectional study included a convenience sample of US residents aged 13 to 40 years who completed an online survey in November and December 2021. Quota sampling was used for an equal proportion of participants aged 13 to 17 years, 18 to 20 years, and 21 to 40 years, balanced for sex, race, and ethnicity per the latest US Census.

**Main Outcomes and Measures:**

Nonnicotine e-cigarette use (ever, past 30- and past 7-day, number of times used, time taken to finish); co-use with nicotine e-cigarettes; age at first try; and flavors, brands, and ingredients used.

**Results:**

Overall, 6131 participants (mean [SD] age, 21.9 [6.8] years; range, 13-40 years; 3454 [56.3%] identifying as female) completed the survey (55.1% completion rate). Among all participants, 1590 (25.9%) had ever used a nonnicotine e-cigarette, 1021 (16.7%) used one in the past 30 days, and 760 (12.4%) used one in the past 7 days. By age group, 227 of 1630 participants aged 13 to 17 years (13.9%), 497 of 2033 participants aged 18 to 20 years (24.4%), 399 of 1041 participants aged 21 to 24 years (38.3%), and 467 of 1427 participants aged 25 to 40 years (32.7%) had ever used nonnicotine e-cigarettes. Among 1590 participants who had ever used a nonnicotine e-cigarette, 549 (34.5%) had used one more than 10 times; 1017 (63.9%) finished 1 nonnicotine e-cigarette in less than 1 week. Co-use of nonnicotine with nicotine e-cigarettes was reported by 1155 participants (18.8%), 1363 (22.2%) exclusively used nicotine e-cigarettes, and 431 (7.0%) exclusively used nonnicotine e-cigarettes. Most-used flavors were sweet, dessert, or candy (578 [36.3%]); fruit (532 [33.4%]); and mint or menthol (321 [20.2%]); similar flavor patterns were observed for the top 2 flavors among those who used nonnicotine e-cigarettes in the past 30 days, followed by combinations of coffee, alcohol, flower, plant, and mint or menthol flavors by age group. Participants most reported using tetrahydrocannabinol (587 [36.9%]), cannabidiol (537 [33.7%]), melatonin (438 [27.5%]), caffeine (428 [26.9%]), and essential oils (364 [22.9%]) in their nonnicotine e-cigarettes.

**Conclusions and Relevance:**

In this study of adolescents, young adults, and adults, a sizeable proportion reported having used nonnicotine e-cigarettes and co-using them with nicotine e-cigarettes. Surveillance studies should further assess nonnicotine e-cigarette use patterns and regulations, and prevention should be developed to address youth appeal, unsubstantiated health claims, and possible health harms.

## Key Points

**Question:**

To what extent are nonnicotine e-cigarettes used, and what flavors, brands, and ingredients are most common?

**Findings:**

This cross-sectional study of 6131 US residents aged 13 to 40 years found that 25.9% had ever used a nonnicotine e-cigarette, 16.7% had used one in the past 30 days, and 12.4% had used one in the past 7 days; 18.8% had ever co-used nonnicotine and nicotine e-cigarettes. The most-used flavors were sweet, dessert, or candy flavors; fruit flavors; and mint or menthol flavors; most common ingredients were tetrahydrocannabinol, cannabidiol, melatonin, caffeine, and essential oils.

**Meaning:**

These findings suggest that a significant proportion of US residents are using nonnicotine flavored e-cigarettes marketed with unsupported health claims, which warrants further research, regulation, and prevention.

## Teaser

This cross-sectional study evaluates nonnicotine e-cigarette use patterns, including common flavors, brands, and ingredients.

## Introduction

Millions of adolescents,^1^ young adults,^2^ and adults^3^ use nicotine e-cigarettes. Research also shows that cannabis use in e-cigarettes is common among youth.^4,5,6,7,8^ While e-cigarettes containing tetrahydrocannabinol (THC) and cannabidiol (CBD) have been on the market for some years, other nonnicotine e-cigarette products have more recently entered the market. However, we lack information about the use of these other nonnicotine e-cigarettes.^9,10^ These products state that they do not contain nicotine; however, the inhaled liquid and/or resulting aerosol contain essential oils, melatonin, caffeine, herbs and herbal supplements, vitamins, flavoring chemicals, and solvents.^5,10,11,12^

Several health and safety concerns that apply to nicotine e-cigarettes may translate to nonnicotine e-cigarettes. Ingredients reported in nonnicotine e-cigarettes (based on websites and packaging) are also present in nicotine e-cigarettes. Some of these ingredients, such as propylene glycol, vegetable glycerin, aldehydes, volatile organic compounds, polycyclic aromatic hydrocarbons, metals, silicate particles, and other heavy metals,^13,14^ are found on the US Food and Drug Administration’s (FDA) list of harmful and potentially harmful constituents in tobacco products.^15^ Aldehydes may increase cancer risk,^16^ flavorants are associated with inflammation and cell dysfunction in in vitro studies,^17,18^ and propylene glycol and vegetable glycerin can disrupt cell metabolism and airway function.^19,20^ Using cannabis is associated with risks of addiction and adverse effects on brain development, especially among youth,^21^ and recent data indicate that inhaled cannabis in e-cigarettes has significantly greater psychological and physiological effects compared with its traditional smoked form.^22^ As in the case of nicotine e-cigarettes, where independent chemical assessments have identified more chemical compounds with toxic health effects than nicotine e-cigarettes claim,^23^ the overall negative effect of harmful or potentially harmful ingredients in nonnicotine e-cigarettes remains unknown.

Research has focused on prevalence,^1,7,24^ device types,^1,25,26^ patterns of use,^7,24,27,28^ and health effects of using e-cigarettes with nicotine^29,30,31,32,33,34^ and cannabis.^8,22^ However, few studies address nonnicotine e-cigarettes, including usage patterns; preferred brands, flavors, and other ingredients; and co-use with nicotine e-cigarettes. Given health concerns regarding nonnicotine e-cigarettes, it is important to understand the extent to which nonnicotine e-cigarettes are being used as well as what brands, flavors, and ingredients are used most. This study examines nonnicotine e-cigarette use among a national online sample of adolescents, young adults, and adults and identifies the specific brands, flavors, and ingredients being used. In addition, given that we know that nicotine e-cigarette use is common, we assess the extent to which people are co-using nonnicotine and nicotine e-cigarettes. Findings from this study can guide further surveillance of nonnicotine e-cigarette use and concurrent use of nonnicotine and nicotine e-cigarettes; identify areas for research on ingredients, flavor types, and health effects from using nonnicotine e-cigarettes; inform targeted and specific prevention messages aimed at reducing use of nonnicotine e-cigarettes; and guide regulation of nonnicotine e-cigarettes that make unsubstantiated health claims or market to youth.

## Methods

In this cross-sectional study, we conducted a national, anonymous online survey from November 17 to December 15, 2021. Participants were recruited by Qualtrics from its network of online research panels; interested panelists provided online consent (for those 18 years and older) or assent (for those younger than 18 years) before beginning the self-administered survey. Per the institutional review board, we were granted a waiver of parental consent for those under age 18 years because the surveys were anonymous, to maintain privacy of participant use patterns and prevent breach of confidentiality regarding potential self-incriminating or antisocial behavior, and to avoid parental coercion. The institutional review board at Stanford University approved the study protocol. This study followed the American Association for Public Opinion Research (AAPOR) reporting guideline by describing the study sample, reporting survey completion rate, and following procedures to maintain data quality.^41^

Our sampling plan was to recruit 2000 adolescents, young adults, and adults (6000 participants total) with the following sampling quotas: 1:1:1 ratio of participants aged 13 to 17 years (adolescents), 18 to 20 years (under the legal age of e-cigarette sales), and 21 to 40 years (young adults and adults) as well as self-reported sex, race, and ethnicity that approximated the US Census. These age groups allow us to address our research questions among a range of people from early adolescence through young adulthood (younger and older than the federal legal age of tobacco sales) to adults who have passed the phase of brain development and are no longer at heightened risk for addiction.^35,36^ Once each quota was met, additional participants representing that subgroup would not be included in the survey. The average time taken to complete a survey was 28 minutes. Participants who completed the survey in less than half of the median completion time and those who failed attention check questions were excluded. Participants could only take the survey once.

### Measures

Survey items were developed and pilot tested with 42 adolescents, young adults, and adults. Sample sociodemographic information included self-reported age, sex (male, female, nonbinary or other, and prefer not to say), gender and sexual orientation (heterosexual or straight, LGBTQ+ [lesbian, gay, bisexual, transgender, questioning, queer, intersex, pansexual, two-spirit (2S), androgynous, or asexual], not listed above [please specify], and prefer not to say), race and ethnicity classified by investigators (Hispanic or Latino; non-Hispanic Asian, Native Hawaiian, or Pacific Islander; non-Hispanic Black or African American; non-Hispanic White; non-Hispanic other or multiracial; and prefer not to say), and financial comfort (do not meet basic expenses, just meet expenses with nothing left over, meet needs with a little left over, live comfortably, and prefer not to say).^37,38^

#### Use of Nicotine e-Cigarettes, Nonnicotine e-Cigarettes, and Combustible Cigarettes

Consistent with survey best practices,^39,40^ all participants first viewed a description of nonnicotine e-cigarettes (eFigure in the Supplement), including different terms used to refer to these products (eg, personal diffusers), example brand names, and accompanying photographs. We then asked all participants, “Have you ever used any of these products in your entire life? (Please select Yes or No)” corresponding to the following products: “(a) Non-nicotine e-cigarettes like Monq or Vitaminvape, even 1 or 2 puffs; (b) Disposable nicotine vapes like Puffbar or FOGG, even 1 or 2 puffs; (c) Pod-based nicotine vapes like JUUL or Phix, even 1 or 2 puffs; and (d) Any other nicotine vape like mods, even 1 or 2 puffs.” Participants selecting “yes” to the first option (ie, a) were noted as having ever used nonnicotine e-cigarettes and those selecting “yes” for the remaining options (ie, b, c, and/or d) were noted as having ever used nicotine e-cigarettes. For any product ever used, we asked participants to indicate past 30-day use (0-30 days) and past 7-day use (0-7 days). For each product, we also asked, “At what age did you first try these products?”

Participants who had ever used a nonnicotine e-cigarette were asked, “How many times in your entire life have you used these products?” “When was the last time you used a vape, even one or two times?” “How soon after you wake up do you use these products?” and “How long does it usually take you to finish 1 nonnicotine vape?” Finally, we asked those who had ever used nonnicotine e-cigarettes, “How likely is it that over the next 6 months you will use a non-nicotine vape again?” with answer choices ranging from 1, indicating very unlikely, to 4, very likely. Participants indicating any response except 1 were considered likely to use in the next 6 months.

#### Ingredients Used in Nonnicotine e-Cigarettes

We asked all participants who had ever used nonnicotine e-cigarettes, “Which of the following were in a non-nicotine e-cigarette you ever used?” There were 9 randomly presented answer choices: THC, CBD, melatonin, caffeine, essential oil, tea, vitamin B, vitamin C, and do not know.

#### Nonnicotine e-Cigarette Brands Used

For 17 randomly presented brands noted in eTable 2 in the Supplement, those who had ever used nonnicotine e-cigarettes were asked, “Which of the following non-nicotine or zero nicotine vapes have you used?” For each brand there were 3 answer choices: (1) never used this brand; (2) used, but not in the past 30 days; and (3) used in the past 30 days.

#### Flavor Types Used in Nonnicotine e-Cigarettes

We asked participants who had ever used a nonnicotine e-cigarette, “Which of these flavors or smells were in any nonnicotine vape that you used? (select all that apply).” There were 18 randomly presented answer choices: (1) flowers (eg, rose, lavender, geranium, jasmine, hibiscus); (2) essential oils (eg, frankincense, tea tree); (3) other plant extract (eg, gingko, sandalwood); (4) spice (eg, clove, cinnamon, nutmeg); (5) coffee or any related flavor (eg, espresso, latte, cappuccino); (6) tea; (7) mint; (8) menthol; (9) ice; (10) wintergreen; (11) fruit (eg, mango, lychee, cherry, blueberry, strawberry, watermelon, coconut); (12) sweets or dessert flavors (eg, crème or crème brûlée, caramel, vanilla, chocolate, ice cream, mud pie); (13) alcohol (eg, wine, bourbon, rum, brandy, tequila, whiskey, beer, Mai Tai, daiquiri); (14) candy; (15) other beverage (eg, cola); (16) unflavored; (17) “don’t know or don’t remember”; and (18) other, please specify (text entry option). Any use of flavor types mint, menthol, ice, or wintergreen was collapsed into mint or menthol flavor type, and flavor types of sweets and candy were collapsed into sweet, dessert, or candy. Next, we asked participants to identify flavors used in the past 30 days and the flavor used most in the past 30 days.

### Statistical Analysis

Descriptive analyses were performed to ascertain nonnicotine e-cigarette usage (never, ever, and past 30- and 7-day use) across sociodemographic factors and co-use with nicotine e-cigarettes. Using a multivariate test of means, we assessed whether the mean age of trying nonnicotine e-cigarettes was equal to that of nicotine e-cigarettes. Next, among those who ever used nonnicotine e-cigarettes, we summarized use-related behaviors and self-reported ingredients by age group (13-17 years, 18-20 years, 21-24 years, and 25-40 years). eTable 1 in the Supplement presents nonnicotine e-cigarette use behaviors among those who used them in the past 30 days. In addition, we reported ever and past 30-day use of nonnicotine e-cigarette brands by age (eTable 2 in the Supplement). We then compiled a list of ingredients per brand, corresponding to all products described on the brand website (eTable 3 in the Supplement). Among those who ever used nonnicotine e-cigarettes, we tabulated flavor types used in nonnicotine e-cigarettes overall and in the past 30 days by age and most-used in the past 30 days. For any given question, responses were missing a maximum of 5% of participants. We report the percentage of missing responses in the footnote following each table. Statistical analysis was conducted in Stata version 15.1 (StataCorp). All tests were 2-tailed, with significance set at *P* < .05.

## Results

Per the AAPOR reporting guidelines, we report that 6131 of 11 118 people who clicked on the survey link completed the entire survey (ie, 55.1% survey completion rate). Among the 11 118 responders, 4306 clicked the survey link after their age, sex, and race and ethnicity quota had been filled and were therefore not included in the final study sample. We further excluded 681 people for not meeting quality and attention checks. The final sample included 6131 participants who completed the entire survey.

Participants’ mean age was 21.9 (SD, 6.8; range, 13-40 years); 3454 (56.3%) self-reported as female, 1573 (25.7%) as LGBTQ+, 318 (5.2%) as Asian, Native Hawaiian, or Pacific Islander non-Hispanic, 841 (13.7%) as Black or African American non-Hispanic, 1143 (18.6%) as Hispanic or Latino, 3162 (51.6%) as White non-Hispanic, 250 (4.1%) as other or multiracial non-Hispanic (including American Indian and Alaska Native non-Hispanic and participants identifying as >1 race non-Hispanic), and 417 (6.8%) preferred not to say (Table 1).

**Table 1.** Participant Characteristics Overall and by Nonnicotine e-Cigarette Use

Characteristic	Participants, No. (%)				
	Total sample (N = 6131)	Never used nonnicotine e-cigarettes (n = 4488)	Ever used nonnicotine e-cigarettes (n = 1590)	Past 30-d use of nonnicotine e-cigarettes (n = 1021)	Past 7-d use of nonnicotine e-cigarettes (n = 760)
Age, y					
13-17	1630 (26.6)	1391 (31.0)	227 (14.3)	113 (11.1)	76 (10.0)
18-20	2033 (33.1)	1519 (33.8)	497 (31.2)	265 (25.9)	198 (26.0)
21-24	1041 (17.0)	632 (14.1)	399 (25.1)	281 (27.5)	208 (27.4)
25-40	1427 (23.3)	946 (21.1)	467 (29.4)	362 (35.5)	278 (36.6)
Sex					
Male	2326 (38.0)	1670 (37.2)	635 (39.9)	446 (43.7)	345 (45.4)
Female	3454 (56.3)	2546 (56.6)	882 (55.5)	541 (53.0)	393 (51.7)
Nonbinary or other	289 (4.7)	227 (5.0)	62 (3.9)	27 (2.6)	18 (2.4)
Prefer not to say	63 (1.0)	52 (1.2)	11 (0.7)	7 (0.7)	4 (0.5)
LGBTQ+					
Yes	1573 (25.7)	1133 (25.2)	432 (27.2)	253 (24.8)	190 (25.0)
No	4251 (69.3)	3125 (69.5)	1091 (68.6)	719 (70.4)	536 (70.5)
Other, not listed above	50 (0.8)	40 (0.9)	9 (0.6)	7 (0.7)	5 (0.7)
Prefer not to say	257 (4.2)	197 (4.4)	58 (3.6)	42 (4.1)	29 (3.8)
Race and ethnicity					
Asian, Native Hawaiian, or Pacific Islander, non-Hispanic	318 (5.2)	254 (5.7)	62 (3.9)	43 (4.2)	34 (4.5)
Black or African American, non-Hispanic	841 (13.7)	575 (12.8)	253 (15.9)	177 (17.4)	133 (17.5)
Hispanic or Latino	1143 (18.6)	698 (15.5)	436 (27.4)	297 (29.1)	225 (29.6)
White, non-Hispanic	3162 (51.6)	2479 (55.2)	661 (41.6)	386 (37.8)	284 (37.4)
Other or multiracial, non-Hispanic	250 (4.1)	191 (4.3)	56 (3.5)	33 (3.2)	24 (3.1)
Prefer not to say	417 (6.8)	291 (6.5)	122 (7.7)	85 (8.3)	60 (7.9)
Financial comfort					
Do not meet basic expenses	314 (5.1)	237 (5.3)	75 (4.7)	35 (3.4)	26 (3.4)
Just meet expenses with nothing left over	1114 (18.2)	839 (18.7)	268 (16.9)	165 (16.2)	122 (16.0)
Meet needs with a little left over	1657 (27.0)	1202 (26.8)	436 (27.4)	279 (27.3)	195 (25.7)
Live comfortably	2579 (42.1)	1811 (40.3)	748 (47.0)	506 (49.6)	398 (52.4)
Prefer not to say	467 (7.6)	399 (8.9)	63 (4.0)	36 (3.5)	19 (2.5)
Nicotine e-cigarette use^a^					
Never-used	3548 (57.9)	3094 (68.9)	431 (27.1)	254 (24.9)	192 (25.2)
Ever used^b^	2536 (41.3)	1363 (30.4)	1155 (72.6)	764 (74.8)	566 (74.5)
Did not answer	47 (0.8)	31 (0.7)	4 (0.3)	3 (0.3)	2 (0.3)
Past 30-d nicotine e-cigarette use^b^	1705 (27.8)	821 (18.3)	871 (54.8)	691 (67.7)	522 (68.7)
Past 7-d nicotine e-cigarette use	1346 (21.9)	638 (14.2)	697 (43.8)	555 (54.3)	471 (62.0)

Abbreviation: LGBTQ+, lesbian, gay, bisexual, transgender, questioning, queer, intersex, pansexual, 2-spirit, androgynous, or asexual.

^a^ Responses were missing for less than 1% of participants who were asked questions about ever use of nicotine e-cigarettes and reported as “Did not answer.”

^b^ Ever use includes past 30-day use, and past 30-day use includes past 7-day use.

### Nonnicotine e-Cigarette Use

Among all participants, 1590 (25.9%) had ever used a nonnicotine e-cigarette; 1021 (16.7%) had used a nonnicotine e-cigarette in the past 30 days, and 760 (12.4%) used a nonnicotine e-cigarette in the past 7 days. Table 1 includes more details on participant characteristics by nonnicotine e-cigarette use.

Among all participants who had ever used a nonnicotine e-cigarette, 549 (34.5%) had used such devices more than 10 times, 294 (18.5%) had used such devices more than 20 times, and 82 (5.2%) had used them more than 100 times. Overall, 469 (29.5%) reported using within 5 minutes of waking up, 605 (38.1%) used it as recently as on the day of the survey, 1017 (63.9%) took less than 1 week to finish 1 nonnicotine e-cigarette, and 1186 (74.6%) were likely to use a nonnicotine e-cigarette again in the next 6 months (Table 2).

**Table 2.** Nonnicotine e-Cigarette Use Behaviors Among 1590 Participants Who Ever Used Them, by Age Group^a^

Characteristic	Participants, No. (%)				
	Ever used nonnicotine e-cigarettes (N = 1590)	Age 13-17 y (n = 227)	Age 18-20 y (n = 497)	Age 21-24 y (n = 399)	Age 25-40 y (n = 467)
No. of times ever used					
1-2 times	629 (39.6)	111 (48.9)	228 (45.9)	144 (36.1)	146 (31.3)
3-10 times	399 (25.1)	44 (19.4)	124 (25.0)	112 (28.1)	119 (25.5)
11-19 times	255 (16.0)	26 (11.4)	65 (13.1)	69 (17.3)	95 (20.3)
20-30 times	159 (10.0)	15 (6.6)	36 (7.2)	40 (10.0)	68 (14.6)
31-99 times	53 (3.3)	15 (6.6)	13 (2.6)	10 (2.5)	15 (3.2)
≥100 times	82 (5.2)	12 (5.3)	25 (5.0)	21 (5.3)	24 (5.1)
Did not answer	13 (0.8)	4 (1.8)	6 (1.2)	3 (0.7)	0
How soon after you wake up do you use					
≤5 min	469 (29.5)	69 (30.4)	151 (30.4)	109 (27.3)	140 (30.0)
6-30 min	434 (27.3)	35 (15.4)	113 (22.7)	127 (31.8)	159 (34.0)
31-60 min	179 (11.2)	12 (5.3)	47 (9.5)	57 (14.3)	63 (13.5)
>60 min	448 (28.2)	93 (41.0)	162 (32.6)	97 (24.3)	96 (20.6)
Did not answer	60 (3.8)	18 (7.9)	24 (4.8)	9 (2.3)	9 (1.9)
Last time vaped					
Earlier today	605 (38.1)	69 (30.4)	176 (35.4)	167 (41.8)	193 (41.4)
Not today, but sometime during the past 7 d	316 (19.9)	35 (15.4)	93 (18.7)	79 (19.8)	109 (23.3)
Not during the past 7 d, but sometime during the past 30 d	151 (9.5)	25 (11.0)	55 (11.1)	41 (10.3)	30 (6.4)
Not during the past 30 d, but sometime during the past 6 mo	110 (6.9)	28 (12.3)	41 (8.2)	24 (6.0)	17 (3.6)
Not during the past 6 mo, but sometime during the past year	156 (9.8)	20 (8.8)	53 (10.7)	37 (9.3)	46 (9.9)
1 to 4 y Ago	139 (8.7)	28 (12.3)	46 (9.3)	22 (5.5)	43 (9.2)
≥5 y Ago	97 (6.1)	17 (7.5)	32 (6.4)	25 (6.3)	23 (4.9)
Did not answer	16 (1.0)	5 (2.2)	1 (0.2)	4 (1.0)	6 (1.3)
How long does it take to finish 1 nonnicotine e-cigarette?					
<1 d	121 (7.6)	14 (6.2)	28 (5.6)	32 (8.0)	47 (10.1)
1-2 d	180 (11.3)	14 (6.2)	42 (8.5)	44 (11.0)	80 (17.1)
3-5 d	356 (22.4)	31 (13.7)	112 (22.5)	92 (23.1)	121 (25.9)
7 d	360 (22.6)	37 (16.3)	117 (23.5)	100 (25.1)	106 (22.7)
2 wk	163 (10.3)	26 (11.4)	46 (9.3)	46 (11.5)	45 (9.6)
1 mo	133 (8.4)	23 (10.1)	51 (10.3)	34 (8.5)	25 (5.4)
“Don’t know”	216 (13.6)	64 (28.2)	87 (17.5)	40 (10.0)	25 (5.4)
Did not answer	61 (3.8)	18 (7.9)	14 (2.8)	11 (2.8)	18 (3.8)
How likely is it that over the next 6 mo you will use a nonnicotine e-cigarette again?^b^	1186 (74.6)	150 (66.1)	340 (68.4)	306 (76.7)	390 (83.5)

^a^ Responses were missing for less than 3.8% of participants who were asked questions about ever use of nonnicotine e-cigarettes and reported as “did not answer.”

^b^ Any response except very unlikely was considered likely to use a nonnicotine e-cigarette in the next 6 months.

Examining nonnicotine e-cigarette use by age group, 227 of 1630 participants aged 13 to 17 years (13.9%), 497 of 2033 aged 18 to 20 years (24.4%), 399 of 1041 aged 21 to 24 years (38.3%), and 467 of 1427 aged 25 to 40 years (32.7%) had ever used nonnicotine e-cigarettes. In the past 30 days, 113 of those aged 13 to 17 years (6.9%); 265 of those aged 18 to 20 years (13.0%), 281 of those aged 21 to 24 years (27.0%), and 362 of those aged 25 to 40 years (25.4%) had used nonnicotine e-cigarettes. Among those younger than age 24, between 16% and 20% reported using more than 20 times, between 27% and 30% reported using within 5 minutes of waking up, between 30% and 40% used as recently as on the day of the survey, and between 66% and 77% were likely to use a nonnicotine e-cigarette again in the next 6 months. Furthermore, between 42% and 67% of nonnicotine e-cigarette ever users younger than 24 years took less than 1 week to finish 1 nonnicotine e-cigarette. Additional information about nonnicotine vaping behaviors among past 30-day users of nonnicotine e-cigarettes is available in eTable 1 in the Supplement.

### Co-use of Nonnicotine and Nicotine e-Cigarettes

In our sample, 2536 participants (41.3%) had ever used a nicotine e-cigarette, 1705 (27.8%) had used in the past 30 days, and 1346 (21.9%) had used in the past 7 days (Table 1). Overall, 1363 participants (22.2%) had exclusively ever used nicotine e-cigarettes, 431 (7.0%) had exclusively ever used nonnicotine e-cigarettes, and 1155 participants (18.8%) had used both nicotine and nonnicotine e-cigarettes. In the past 30 days, 176 participants (2.9%) had exclusively used nicotine e-cigarettes, 47 (0.8%) had exclusively used nonnicotine e-cigarettes, and 691 (11.3%) had used both nicotine and nonnicotine e-cigarettes. On average, participants reported first trying non-nicotine e-cigarettes at a younger age (mean [SD], 15.0 [5.1] years) compared with nicotine e-cigarettes, including disposables (mean [SD] age, 16.1 [5.3] years; *P* = .004), pod- or cartridge-based devices (mean [SD] age, 16.3 [5.5] years; *P* < .001), and other e-cigarettes (mean [SD] age, 16.2 [5.3] years; *P* < .001).

### Ingredients Ever Used in Nonnicotine e-Cigarettes

In the total sample, participants most commonly reported having ever used the following ingredients in nonnicotine e-cigarettes: THC (587 [36.9%]), CBD (537 [33.7%]), melatonin (438 [27.5%]), caffeine (428 [26.9%]), and essential oils (364 [22.9%]), in that order (Table 3). Among participants younger than 18 years, most reported using THC (61 [26.9%]) or melatonin (61 [26.9%]), followed by CBD (55 [24.2%]) or essential oils (55 [24.2%]). Among young adults (age 18-20 years), THC (183 [36.8%]) and CBD (158 [31.8%]) were the most commonly reported, followed by melatonin (144 [29.0%]). Young adults (age 21-24 years) followed a similar pattern, with most reporting using THC (158 [39.6%]), CBD (156 [39.1%]) or melatonin (122 [30.6%]). Among adults aged 25 to 40 years, ingredients reportedly used most widely included THC (185 [39.6%]), CBD (168 [36.0%]), and caffeine (145 [31.0%]).

**Table 3.** Self-reported Ingredients in Nonnicotine e-Cigarettes Among 1590 Participants Who Ever Used Them, by Age Group^a^

Ingredient	Participants, No. (%)				
	Total (N = 1590)	Age 13-17 y (n = 227)	Age 18-20 y (n = 497)	Age 21-24 y (n = 399)	Age 25-40 y (n = 467)
THC	587 (36.9)	61 (26.9)	183 (36.8)	158 (39.6)	185 (39.6)
CBD	537 (33.7)	55 (24.2)	158 (31.8)	156 (39.1)	168 (36.0)
Melatonin	438 (27.5)	61 (26.9)	144 (29.0)	122 (30.6)	111 (23.8)
Caffeine	428 (26.9)	44 (19.4)	122 (24.5)	117 (29.3)	145 (31.0)
Essential oil	364 (22.9)	55 (24.2)	104 (20.9)	111 (27.8)	94 (20.1)
Tea	287 (18.0)	28 (12.3)	91 (18.3)	76 (19.0)	92 (19.7)
Vitamin B	203 (12.7)	27 (11.9)	56 (11.3)	52 (13.0)	68 (14.6)
Vitamin C	186 (11.7)	31 (13.7)	49 (9.9)	48 (12.0)	58 (12.4)
Do not know	281 (17.7)	55 (24.2)	96 (19.3)	71 (17.8)	59 (12.6)

Abbreviations: CBD, cannabidiol; THC, tetrahydrocannabinol.

^a^ Participants could select multiple options.

### Nonnicotine e-Cigarette Brands Used

As seen in eTable 2 in the Supplement, Cloudy, Vitaminvape, and Breathe were the most widely ever used brands; in the past 30 days, Vitaminvape was the most-used nonnicotine e-cigarette (242 [23.7%]), followed by Cloudy (233 [22.8%]), Fum (222 [21.7%]), and Ripple+ and HealthVape (220 [21.5%] each). A list of ingredients in 14 nonnicotine e-cigarette brands is provided in eTable 3 in the Supplement, which include vitamin B12, lavender, essential oils, flavoring extracts (eg, orange, peppermint), vegetable glycerin, and propylene glycol.

### Flavor Types Used in Nonnicotine e-Cigarettes

Among those who ever used a nonnicotine e-cigarette, 578 (36.3%) reported ever having used sweet, dessert, or candy flavors; 532 (33.4%) used fruit flavors; and 321 (20.2%) used mint or menthol flavors, closely followed by coffee, flower, and essential oil flavors (Table 4). Among nonnicotine e-cigarette users in the past 30 days, sweet, dessert, and candy flavors (age 13-17 years: 38 [33.6%]; age 18-20 years: 110 [41.5%]; age 21-24 years: 120 [42.7%]; and age 25-40 years: 140 [38.7%]), followed by fruit flavors (age 13-17 years: 33 [29.2%]; age 18-20 years: 74 [27.9%]; age 21-24 years: 99 [35.2%]; and age 25-40 years: 98 [27.1%]) were the most widely used across all age groups; other flavor types varied by age group. After sweet, dessert, and candy flavors and fruit flavors, those aged 13 to 17 years using nonnicotine e-cigarettes in the past 30 days reported using flower (23 [20.4%]), mint or menthol (20 [17.7%]), and other plant extract (17 [15.0%]) flavor types; those aged 18 to 20 years old reported using coffee (52 [19.6%]), flower (46 [17.4%]), and mint or menthol (40 [15.1%]) flavor types; those aged 21 to 24 years reported using alcohol (61 [21.7%]), coffee (57 [20.3%]), and flower (52 [18.5%]) flavor types; and those aged 25 to 40 years reported using alcohol (79 [21.8%]), coffee (77 [21.3%]), and mint or menthol (61 [16.9%]) flavor types. Overall, the most-used flavor types in the past 30 days were sweet, dessert, candy (204 [20.3%]), followed by fruit (170 [16.9%]) and mint or menthol (158 [15.7%]).

**Table 4.** Flavor Types Used in Nonnicotine e-Cigarettes Among Those Who Ever Used And Used in Past 30 Days, by Age^a^

Flavor	Participants, No. (%)										
	Flavors ever used, No. (%)					Flavors used in past 30 d, No. (%)					Most-used flavor in past 30 d (n = 1003)
	Total (n = 1590)	13-17 y (n = 227)	18-20 y (n = 497)	21-24 y (n = 399)	25-40 y (n = 467)	Total (n = 1021)	13-17 y (n = 113)	18-20 y (n = 265)	21-24 y (n = 281)	25-40 y (n = 362)	
Sweet, dessert, or candy											
Any	578 (36.3)	83 (36.6)	178 (35.8)	143 (35.8)	174 (37.3)	408 (40.0)	38 (33.6)	110 (41.5)	120 (42.7)	140 (38.7)	204 (20.3)
Sweet or dessert flavors	425 (26.7)	62 (27.3)	125 (25.2)	114 (28.6)	124 (26.6)	303 (29.7)	25 (22.1)	83 (31.3)	90 (32.0)	105 (29.0)	148 (14.8)
Candy	272 (17.1)	48 (21.1)	81 (16.3)	62 (15.5)	81 (17.3)	163 (16.0)	19 (16.8)	40 (15.1)	52 (18.5)	52 (14.4)	56 (5.6)
Fruit	532 (33.4)	74 (32.6)	171 (34.4)	151 (37.8)	136 (29.1)	304 (29.8)	33 (29.2)	74 (27.9)	99 (35.2)	98 (27.1)	170 (16.9)
Mint or menthol											
Any	321 (20.2)	54 (23.8)	94 (18.9)	83 (20.8)	90 (19.3)	162 (15.9)	20 (17.7)	40 (15.1)	41 (14.6)	61 (16.9)	158 (15.7)
Mint, including spearmint	276 (17.3)	51 (22.5)	81 (16.3)	64 (16.0)	80 (17.1)	134 (13.1)	20 (17.7)	30 (11.3)	34 (12.1)	50 (13.8)	51 (5.1)
Wintergreen	198 (12.4)	26 (11.5)	63 (12.7)	56 (14.0)	53 (11.3)	121 (11.8)	15 (13.3)	29 (10.9)	38 (13.5)	39 (10.8)	36 (3.6)
Menthol	205 (12.9)	24 (10.6)	64 (12.9)	55 (13.8)	62 (13.3)	129 (12.6)	7 (6.2)	40 (15.1)	30 (10.7)	52 (14.4)	40 (4.0)
Ice	167 (10.5)	25 (11.0)	49 (9.9)	46 (11.5)	47 (10.1)	90 (8.8)	9 (8.0)	23 (8.7)	23 (8.2)	35 (9.7)	31 (3.1)
Coffee or any related flavor	287 (18.0)	29 (12.8)	86 (17.3)	81 (20.3)	91 (19.5)	202 (19.8)	16 (14.2)	52 (19.6)	57 (20.3)	77 (21.3)	66 (6.6)
Flowers (eg, lavender)	283 (17.8)	32 (14.1)	82 (16.5)	92 (23.1)	77 (16.5)	181 (17.7)	23 (20.4)	46 (17.4)	52 (18.5)	60 (16.6)	55 (5.5)
Essential oils (eg, tea tree)	245 (15.4)	37 (16.3)	83 (16.7)	65 (16.3)	60 (12.8)	147 (14.4)	11 (9.7)	39 (14.7)	47 (16.7)	50 (13.8)	48 (4.7)
Alcohol	239 (15.0)	21 (9.3)	67 (13.5)	72 (18.0)	79 (16.9)	206 (20.2)	16 (14.2)	50 (18.9)	61 (21.7)	79 (21.8)	76 (7.6)
Other beverage	207 (13.0)	25 (11.0)	54 (10.9)	70 (17.5)	58 (12.4)	148 (14.5)	11 (9.7)	45 (17.0)	44 (15.7)	48 (13.3)	33 (3.3)
Unflavored	181 (11.4)	24 (10.6)	57 (11.5)	48 (12.0)	52 (11.1)	114 (11.2)	12 (10.6)	31 (11.7)	28 (10.0)	43 (11.9)	26 (2.6)
Spice	177 (11.1)	24 (10.6)	48 (9.7)	51 (12.8)	54 (11.6)	150 (14.7)	12 (10.6)	43 (16.2)	41 (14.6)	54 (14.9)	48 (4.8)
Other plant extract	157 (9.9)	20 (8.8)	50 (10.1)	51 (12.8)	36 (7.7)	134 (13.1)	17 (15.0)	39 (14.7)	33 (11.7)	45 (12.4)	32 (3.2)
Tea	148 (9.3)	21 (9.3)	31 (6.2)	45 (11.3)	51 (10.9)	100 (9.8)	11 (9.7)	25 (9.4)	21 (7.5)	43 (11.9)	31 (3.1)
Do not know or do not remember	164 (10.3)	35 (15.4)	61 (12.3)	35 (8.8)	33 (7.1)	81 (7.9)	16 (14.2)	24 (9.1)	18 (6.4)	23 (6.4)	44 (4.4)
Other	38 (2.4)	6 (2.6)	10 (2.0)	10 (2.5)	12 (2.6)	24 (2.3)	4 (3.5)	10 (3.8)	7 (2.5)	3 (0.8)	12 (1.1)

^a^ Participants could select multiple flavor types (not mutually exclusive categories) for both flavors ever used, and flavors used in the past 30 days; most-used flavors in the past 30 days were reported as mutually exclusive categories.

## Discussion

This study extends the limited literature on nonnicotine e-cigarette products, which has largely focused on similarities with nicotine e-cigarette marketing, on toxic effects from what the industry refers to as vitamin vaping,^9,10^ or on just cannabis (THC or CBD) e-cigarette use.^5,6,7,8,22^ Our findings show that nonnicotine e-cigarettes are commonly used across all age groups, including in the past 30 and past 7 days, soon after waking, and as recently as on the day of the survey. Furthermore, there was substantial co-use of nonnicotine and nicotine e-cigarettes, with participants first trying nonnicotine e-cigarettes on average at a younger age than nicotine e-cigarettes. Overall, the most common ingredients used in nonnicotine e-cigarettes were THC, CBD, melatonin, and caffeine. Cloudy and Vitaminvape were the most popular and commonly used nonnicotine e-cigarette brands. The most-used flavor types among all participants in the past 30 days were sweet, dessert, or candy; fruit; caffeine; alcohol; flower; and mint or menthol flavors.

Although no prior data on the specifics of nonnicotine e-cigarette use are, to our knowledge, available, findings from studies on nicotine e-cigarette products and use patterns can inform and support our findings. First, like findings that disposable nicotine e-cigarettes are now the most widely used nicotine e-cigarette,^1,26^ nearly all brands of nonnicotine e-cigarettes resemble disposable-type devices. Second, similar to findings that adolescent nicotine e-cigarette users report using multiple device types,^1,26^ we found substantial co-use of nonnicotine and nicotine e-cigarettes. Third, similar to nicotine e-cigarette studies,^42^ many users and especially young adults do not know what ingredients or flavors they are using in their nonnicotine e-cigarettes.^43^ Finally, the most commonly used flavors in nonnicotine e-cigarettes in the past 30 days were fruit and sweet, dessert, candy flavors, similar to most commonly used flavors in nicotine^1^ and cannabis^44^ e-cigarettes.

Our study highlights several issues of concern to researchers and policy makers. First, although nonnicotine e-cigarettes share many of the same ingredients as nicotine e-cigarettes, including several associated with known health harms, the health effects of aerosolizing and inhaling chemicals such as essential oils, vitamins, caffeine, and other herbs remain unknown.^13,19,20^ There may be risks in using nonnicotine e-cigarettes alone and additive risk to those co-using with nicotine e-cigarettes. Several nonnicotine e-cigarette companies seem to acknowledge harms and suggest that people should not inhale the aerosol into their lungs (eg, one website recommends breathing into the mouth and gently out through the nose).^45^ It remains unknown whether users of these devices are using these products as intended. Second, the FDA has raised concerns in warning letters to nonnicotine e-cigarette companies and public advisories, noting that while several ingredients may be “generally recognized as safe” and thus approved as dietary supplements for ingestion, they may be harmful when inhaled in aerosolized form and cannot be legally marketed as dietary supplements.^46,47^ Third, nonnicotine e-cigarette companies appear to be targeting adolescents and young adults by marketing their products with unproven health benefits, such as an improved immune system, weight loss, better sleep, and more energy,^10,11,45,48^ which may appeal to adolescents’ and young adults’ needs to appear trendy, environmentally friendly, and tech-savvy and to prioritize self-care. Finally, nonnicotine e-cigarettes are currently not regulated as either tobacco products^49^ or dietary supplements.^50^ With aggressive marketing targeting young people and gaps in regulation, the popularity and use of nonnicotine e-cigarettes can be expected to increase dramatically, just as nicotine e-cigarette use grew exponentially between 2015 and 2019.^51^ Thus, rapid surveillance of nonnicotine e-cigarette use is needed to accurately and continually estimate use patterns and health harms associated with these products as well as to develop regulatory solutions. In so doing, it is important to ask about specific brand names, flavors, and ingredients, given that evidence suggests that asking about specific vaping devices and e-cigarette content is more sensitive than asking about general use.^39^

### Limitations

This study has several limitations. First, we report findings from a convenience sample, and thus the rates of use provided in our study are restricted to our sample and may not be generalizable. Second, self-reported information about participants’ use of products may be subject to recall-related errors. Third, participants were not asked about specific products used within each brand, which may have helped to identify which ingredients participants used instead of a broader question about ingredients used. Fourth, we cannot ascertain whether participants understood the difference between CBD and THC or if they misclassified their cannabis use.

## Conclusions

In this study, a sizeable number of adolescents, young adults, and adults reported having used nonnicotine e-cigarettes and co-using them with nicotine e-cigarettes. Despite unproven health and wellness claims made about these products, their safety is unknown and may present health harms from inhaling flavorants and other constituents. National surveillance studies should be conducted to further understand nonnicotine e-cigarette patterns and to develop regulations that address possible health harms of nonnicotine e-cigarettes. Our findings also suggest that the FDA should enforce against nonnicotine e-cigarettes that are making unsubstantiated dietary or other unauthorized health claims and targeting adolescent and young adult customers.

## Notes

Supplement. **eFigure. **Description of Nonnicotine e-Cigarettes in the Survey **eTable 1. **Nonnicotine e-Cigarette–Related Behaviors Among Past 30-Day Users of Nonnicotine Vapes, by Age **eTable 2. **Brands Used Among Ever Users (n=1590) and Past 30-Day Users (n=1021) of Nonnicotine e-Cigarettes, by Age **eTable 3. **Reported Ingredients in Nonnicotine e-Cigarettes, by Brand and Product

## References

[1] Park-Lee E, Ren C, Sawdey MD, . Notes from the field: e-cigarette use among middle and high school students—National Youth Tobacco Survey, United States, 2021. MMWR Morb Mortal Wkly Rep. 2021;70(39):1387-1389. doi:10.15585/mmwr.mm7039a4 34591834 PMC8486384

[2] US Department of Health and Human Services. e-Cigarette use among youth and young adults: a report of the surgeon general. 2016. Accessed April 25, 2022. https://www.cdc.gov/tobacco/data_statistics/sgr/e-cigarettes/index.htm

[3] Creamer MR, Wang TW, Babb S, . Tobacco product use and cessation indicators among adults—United States, 2018. MMWR Morb Mortal Wkly Rep. 2019;68(45):1013-1019. doi:10.15585/mmwr.mm6845a2 31725711 PMC6855510

[4] Watson CV, Puvanesarajah S, Trivers KF. Racial and ethnic differences in marijuana use in e-cigarettes among US youth in 2017, 2018, and 2020. JAMA Pediatr. 2021;175(7):746-748. doi:10.1001/jamapediatrics.2021.0305 33900395 PMC8077037

[5] Trivers KF, Gentzke AS, Phillips E, Tynan M, Marynak KL, Schauer GL. Substances used in electronic vapor products among adults in the United States, 2017. Addict Behav Rep. 2019;10:100222. doi:10.1016/j.abrep.2019.100222 31828201 PMC6888746

[6] Dai H. Self-reported marijuana use in electronic cigarettes among US youth, 2017 to 2018. JAMA. 2020;323(5):473-474. doi:10.1001/jama.2019.19571 31848567 PMC6990743

[7] Miech R, Leventhal A, Johnston L, O’Malley PM, Patrick ME, Barrington-Trimis J. Trends in use and perceptions of nicotine vaping among US youth from 2017 to 2020. JAMA Pediatr. 2021;175(2):185-190. doi:10.1001/jamapediatrics.2020.5667 33320241 PMC7739194

[8] Pearson JL, Villanti AC. It is past time to consider cannabis in vaping research. Nicotine Tob Res. 2020;22(5):597-598. doi:10.1093/ntr/ntaa012 31956918 PMC7171266

[9] Sultan AS, Jessri M, Farah CS. Electronic nicotine delivery systems: oral health implications and oral cancer risk. J Oral Pathol Med. 2021;50(3):316-322. doi:10.1111/jop.12810 30507043

[10] Basáñez T, Majmundar A, Cruz TB, Allem J-P, Unger JB. e-Cigarettes are being marketed as “vitamin delivery” devices. Am J Public Health. 2019;109(2):194-196. doi:10.2105/AJPH.2018.304804 30649935 PMC6336045

[11] Cloudy. Accessed December 29, 2021. https://trycloudy.com/

[12] Ripple+. Accessed April 13, 2022. https://www.therippleco.com/collections/new-shop/products/focus

[13] Pisinger C, Døssing M. A systematic review of health effects of electronic cigarettes. Prev Med. 2014;69:248-260. doi:10.1016/j.ypmed.2014.10.009 25456810

[14] World Health Organization. Electronic nicotine delivery systems and electronic non-nicotine delivery systems (ENDS/ENNDS). 2016. Accessed February 22, 2022. https://escholarship.org/uc/item/2f65f2j5

[15] US Food and Drug Administration. Harmful and potentially harmful constituents in tobacco products and tobacco smoke; established list. April 3, 2012. Accessed December 29, 2021. https://www.federalregister.gov/documents/2012/04/03/2012-7727/harmful-and-potentially-harmful-constituents-in-tobacco-products-and-tobacco-smoke-established-list

[16] Jensen RP, Luo W, Pankow JF, Strongin RM, Peyton DH. Hidden formaldehyde in e-cigarette aerosols. N Engl J Med. 2015;372(4):392-394. doi:10.1056/NEJMc1413069 25607446

[17] Fetterman JL, Weisbrod RM, Feng B, . Flavorings in tobacco products induce endothelial cell dysfunction. Arterioscler Thromb Vasc Biol. 2018;38(7):1607-1615. doi:10.1161/ATVBAHA.118.311156 29903732 PMC6023725

[18] Muthumalage T, Prinz M, Ansah KO, Gerloff J, Sundar IK, Rahman I. Inflammatory and oxidative responses induced by exposure to commonly used e-cigarette flavoring chemicals and flavored e-liquids without nicotine. Front Physiol. 2018;8:1130. doi:10.3389/fphys.2017.01130 29375399 PMC5768608

[19] Woodall M, Jacob J, Kalsi KK, . e-Cigarette constituents propylene glycol and vegetable glycerin decrease glucose uptake and its metabolism in airway epithelial cells in vitro. Am J Physiol Lung Cell Mol Physiol. 2020;319(6):L957-L967. doi:10.1152/ajplung.00123.2020 32996783 PMC7792687

[20] Ghosh A, Coakley RC, Mascenik T, . Chronic e-cigarette exposure alters the human bronchial epithelial proteome. Am J Respir Crit Care Med. 2018;198(1):67-76. doi:10.1164/rccm.201710-2033OC 29481290 PMC6034122

[21] Volkow ND, Baler RD, Compton WM, Weiss SR. Adverse health effects of marijuana use. N Engl J Med. 2014;370(23):2219-2227. doi:10.1056/NEJMra1402309 24897085 PMC4827335

[22] Miech RA, Patrick ME, O’Malley PM, Johnston LD, Bachman JG. Trends in reported marijuana vaping among US adolescents, 2017-2019. JAMA. 2020;323(5):475-476. doi:10.1001/jama.2019.20185 31848566 PMC6990699

[23] Omaiye EE, McWhirter KJ, Luo W, Pankow JF, Talbot P. High-nicotine electronic cigarette products: toxicity of JUUL fluids and aerosols correlates strongly with nicotine and some flavor chemical concentrations. Chem Res Toxicol. 2019;32(6):1058-1069. doi:10.1021/acs.chemrestox.8b00381 30896936 PMC6579667

[24] Dai H, Leventhal AM. Prevalence of e-cigarette use among adults in the United States, 2014-2018. JAMA. 2019;322(18):1824-1827. doi:10.1001/jama.2019.15331 31524940 PMC6749536

[25] Leventhal AM, Dai H, Barrington-Trimis JL, Tackett AP, Pedersen ER, Tran DD. Disposable e-cigarette use prevalence, correlates, and associations with previous tobacco product use in young adults. Nicotine Tob Res. 2022;24(3):372-379. 34379787 10.1093/ntr/ntab165 PMC8842396

[26] Gaiha SM, Lempert LK, McKelvey K, Halpern-Felsher B. e-Cigarette devices, brands, and flavors attract youth: informing FDA’s policies and priorities to close critical gaps. Addict Behav. 2022;126:107179. doi:10.1016/j.addbeh.2021.107179 34861522 PMC8712419

[27] Gaiha SM, Lempert LK, Halpern-Felsher B. Underage youth and young adult e-cigarette use and access before and during the coronavirus disease 2019 pandemic. JAMA Netw Open. 2020;3(12):e2027572. doi:10.1001/jamanetworkopen.2020.27572 33270127 PMC7716191

[28] Pérez A, Bluestein MA, Kuk AE, Chen B. Age of e-cigarette initiation in USA young adults: findings from the Population Assessment of Tobacco and Health (PATH) study (2013-2017). PLoS One. 2021;16(12):e0261243. doi:10.1371/journal.pone.0261243 34898629 PMC8668126

[29] Alzahrani T, Pena I, Temesgen N, Glantz SA. Association between electronic cigarette use and myocardial infarction. Am J Prev Med. 2018;55(4):455-461. doi:10.1016/j.amepre.2018.05.004 30166079 PMC6208321

[30] Ghosh A, Beyazcicek O, Davis ES, Onyenwoke RU, Tarran R. Cellular effects of nicotine salt-containing e-liquids. J Appl Toxicol. 2021;41(3):493-505. doi:10.1002/jat.4060 33034066

[31] Gotts JE, Jordt SE, McConnell R, Tarran R. What are the respiratory effects of e-cigarettes? BMJ. 2019;366:l5275. doi:10.1136/bmj.l5275 31570493 PMC7850161

[32] Kuntic M, Oelze M, Steven S, . Short-term e-cigarette vapour exposure causes vascular oxidative stress and dysfunction: evidence for a close connection to brain damage and a key role of the phagocytic NADPH oxidase (NOX-2). Eur Heart J. 2020;41(26):2472-2483. doi:10.1093/eurheartj/ehz772 31715629 PMC7340357

[33] Riehm KE, Young AS, Feder KA, . Mental health problems and initiation of e-cigarette and combustible cigarette use. Pediatrics. 2019;144(1):e20182935. doi:10.1542/peds.2018-2935 31160343 PMC6615573

[34] Vogel EA, Cho J, McConnell RS, Barrington-Trimis JL, Leventhal AM. Prevalence of electronic cigarette dependence among youth and its association with future use. JAMA Netw Open. 2020;3(2):e1921513-e1921513. doi:10.1001/jamanetworkopen.2019.21513 32074292 PMC7780897

[35] Casey BJ, Jones RM. Neurobiology of the adolescent brain and behavior: implications for substance use disorders. J Am Acad Child Adolesc Psychiatry. 2010;49(12):1189-1201. doi:10.1097/00004583-201012000-00005 21093769 PMC3099425

[36] Yuan M, Cross SJ, Loughlin SE, Leslie FM. Nicotine and the adolescent brain. J Physiol. 2015;593(16):3397-3412. doi:10.1113/JP270492 26018031 PMC4560573

[37] Vallone DM, Cuccia AF, Briggs J, Xiao H, Schillo BA, Hair EC. Electronic cigarette and JUUL use among adolescents and young adults. JAMA Pediatr. 2020;174(3):277-286. doi:10.1001/jamapediatrics.2019.5436 31961395 PMC6990671

[38] Mathur Gaiha S, Halpern-Felsher B, Feld AL, Gaber J, Rogers T, Henriksen L. JUUL and other e-cigarettes: socio-demographic factors associated with use and susceptibility in California. Prev Med Rep. 2021;23:101457. doi:10.1016/j.pmedr.2021.101457 34194963 PMC8227835

[39] Morean ME, Camenga DR, Bold KW, . Querying about the use of specific e-cigarette devices may enhance accurate measurement of e-cigarette prevalence rates among high school students. Nicotine Tob Res. 2020;22(5):833-837. doi:10.1093/ntr/nty240 30395344 PMC7171282

[40] Hair EC, Jodie Briggs M, Thakkar RR, Vallone DM. What pediatric providers need to know about JUUL and other e-cigarettes. J Med Pract Manage: MPM. 2019;34(6):342-343.

[41] American Association for Public Opinion Research. Standard definitions: final dispositions of case codes and outcome rates for surveys. 2016. Accessed April 25, 2022. https://www.aapor.org/aapor_main/media/publications/standard-definitions20169theditionfinal.pdf

[42] McKelvey K, Halpern-Felsher B. How and why California young adults are using different brands of pod-type electronic cigarettes in 2019: implications for researchers and regulators. J Adolesc Health. 2020;67(1):46-52. doi:10.1016/j.jadohealth.2020.01.017 32192827 PMC7311231

[43] Keamy-Minor E, McQuoid J, Ling PM. Young adult perceptions of JUUL and other pod electronic cigarette devices in California: a qualitative study. BMJ Open. 2019;9(4):e026306. doi:10.1136/bmjopen-2018-026306 30948599 PMC6500190

[44] Werts M, Urata J, Watkins SL, Chaffee BW. Flavored cannabis product use among adolescents in California. Prev Chronic Dis. 2021;18:E54. doi:10.5888/pcd18.210026 34081578 PMC8220961

[45] Monq Aromatherapy. Accessed December 29, 2021. https://monq.com/

[46] US Food and Drug Administration. FDA takes action to protect consumers from vaping products with unproven health claims. 2021. Accessed February 22, 2022. https://www.fda.gov/consumers/health-fraud-scams/fda-takes-action-protect-consumers-vaping-products-unproven-health-claims

[47] Food and Drug Administration. Beware of vaping products with unproven health claims. December 7, 2021. Accessed February 22, 2022. https://www.fda.gov/consumers/consumer-updates/beware-vaping-products-unproven-health-claims

[48] Inhale Health. Accessed December 29, 2021. https://www.inhalehealth.com/?utm_source=google&utm_medium=adwords&gclid=Cj0KCQiAq7COBhC2ARIsANsPATHc0n3C2ZkKtQycejgqhkRUvuOt7TJ2I_PuoaU3hqTK62yD9nEshOkaAjBmEALw_wcB

[49] Food and Drug Administration. Deeming tobacco products to be subject to the federal Food, Drug, and Cosmetic Act, as amended by the Family Smoking Prevention and Tobacco Control Act; restrictions on the sale and distribution of tobacco products and required warning statements for tobacco products. May 20, 2016. Accessed February 23, 2022. https://www.federalregister.gov/documents/2016/05/10/2016-10685/deeming-tobacco-products-to-be-subject-to-the-federal-food-drug-and-cosmetic-act-as-amended-by-the 27192730

[50] Dietary Supplement Health and Education Act of 1994, Pub L No. 103-417, 108 Stat 4325. Accessed April 25, 2022. https://ods.od.nih.gov/About/DSHEA_Wording.aspx

[51] Cullen KA, Ambrose BK, Gentzke AS, Apelberg BJ, Jamal A, King BA. Notes from the field: use of electronic cigarettes and any tobacco product among middle and high school students—United States, 2011-2018. MMWR Morb Mortal Wkly Rep. 2018;67(45):1276-1277. doi:10.15585/mmwr.mm6745a5 30439875 PMC6290807

