# Supplementary material for: Use Patterns, Flavors, Brands, and Ingredients of Nonnicotine e-Cigarettes Among Adolescents, Young Adults, and Adults in the United States
Source: JAMA Netw Open. 2022 May 25;5(5):e2216194. doi: 10.1001/jamanetworkopen.2022.16194 (PMC9133942; doi:10.1001/jamanetworkopen.2022.16194)
Supplement: Supplement. — eFigure. Description of Nonnicotine e-Cigarettes in the Survey eTable 1. Nonnicotine e-Cigarette–Related Behaviors Among Past 30-Day Users of Nonnicotine Vapes, by Age eTable 2. Brands Used Among Ever Users (n=1590) and Past 30-Day Users (n=1021) of Nonnicotine e-Cigarettes, by Age eTable 3. Reported Ingredients in Nonnicotine e-Cigarettes, by Brand and Product [file jamanetwopen-e2216194-s001.pdf]

Supplemental Online Content

Gaiha SM, Lin C, Lempert LK, Halpern-Felsher B. Use patterns, flavors, brands, and ingredients of nonnicotine e-cigarettes among adolescents, young adults, and adults in the United States. *JAMA Netw Open.* 2022;5(5):e2216194.
doi:10.1001/jamanetworkopen.2022.16194

eFigure. Description of Nonnicotine e-Cigarettes in the Survey

eTable 1. Nonnicotine e-Cigarette–Related Behaviors Among Past 30-Day Users of Nonnicotine Vapes, by Age

eTable 2. Brands Used Among Ever Users (n=1590) and Past 30-Day Users (n=1021) of Nonnicotine e-Cigarettes, by Age

eTable 3. Reported Ingredients in Nonnicotine e-Cigarettes, by Brand and Product

This supplemental material has been provided by the authors to give readers additional information about their work.

© 2022 Gaiha SM et al. *JAMA Network Open.*

eFigure. Description of Nonnicotine e-Cigarettes in the Survey

Product descriptions
It's important to us that when you answer questions, you are referring to the same products as us, so please read the next few product descriptions and carefully look at accompanying images.

When we show you products or product images, we do not endorse their safety or that you use any of them.

Page Break

Q15

These are examples of products that claim to be **zero nicotine or non-nicotine vapes,** sometimes called health vapes, wellness vapes, healing vapes, therapeutic vapes, energy vapes, plant-based vapes, vitamin vapes, sleep or melatonin vapes, relaxation vapes, herbal vapes, caffeine vapes, essential oil vapes, personal diffuser sticks, and aromatherapy vape pens. Some brands for non-nicotine vapes include ***Monq, Vitaminvape, Nutrovape, Eagle Energy, Breathe, Moxe, Ripple, Cloudy, Teatime, LUVV, Inhale Health and Vitastik***. We will refer to these throughout the survey as "**non-nicotine vapes**."

These are examples of products that claim to be **zero nicotine or non-nicotine vapes,** sometimes called health, therapeutic, energy, vitamin, sleep, relaxation, herbal, caffeine, aromatherapy or diffuser vapes. Some brands for non-nicotine vapes include **Monq, Vitaminvape, Nutrovape, Eagle Energy, Breathe, Moxe, Ripple, Cloudy, Teatime, LUVV, Inhale Health and Vitastik**. We will refer to these throughout the survey as "non-nicotine vapes."

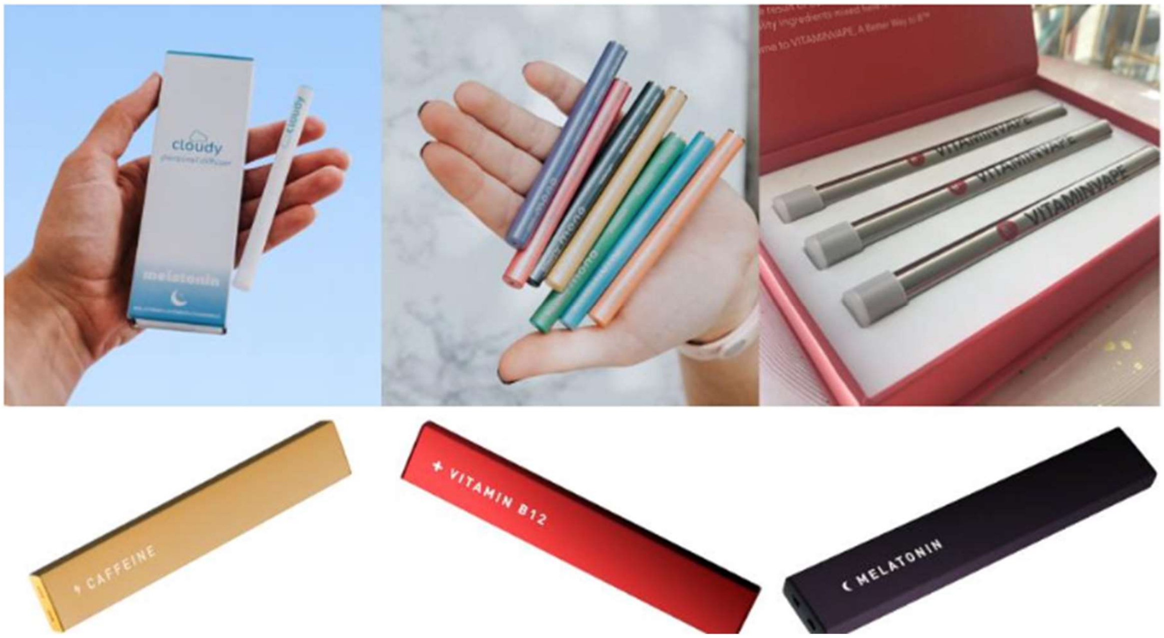

© 2022 Gaiha SM et al. *JAMA Network Open.*

eTable 1. Nonnicotine e-Cigarette–Related Behaviors Among Past 30-Day Users of Nonnicotine Vapes, by Age (N= 1021) [n (%)]

	Past 30-day users of non-nicotine vapes (N = 1,021)	13-17 years (N = 113)	18-20 years (N = 265)	21-24 years (N=281)	25-40 years (N = 362)
Number of times ever used					
1-2 times	305 (29.9)	38 (33.6)	92 (34.7)	81 (28.9)	94 (26.0)
3-10 times	276 (27.0)	28 (24.9)	71 (26.8)	86 (30.6)	91 (25.1)
11-19 times	207 (20.3)	18 (15.9)	49 (18.5)	55 (19.6)	85 (23.5)
20-30 times	129 (12.6)	11 (9.7)	26 (9.8)	33 (11.7)	59 (16.3)
31-99 times	43 (4.2)	11 (9.7)	11 (4.2)	9 (3.2)	12 (3.3)
100 or more times	61 (6.0)	7 (6.2)	16 (6.0)	17 (6.0)	21 (5.8)
How soon after you wake up do you use					
Within 5 minutes	312 (30.5)	36 (31.9)	90 (33.9)	82 (29.2)	104 (28.7)
6-30 minutes	380 (37.2)	29 (25.7)	93 (35.1)	113 (40.2)	145 (40.1)
31-60 minutes	151 (14.8)	12 (10.6)	39 (14.7)	43 (15.3)	57 (15.7)
After 60 minutes	162 (15.9)	33 (29.2)	38 (14.3)	40 (14.2)	51 (14.1)
Did not answer[a]	16 (1.6)	3 (2.6)	5 (2.0)	3 (1.1)	5 (1.4)
Last time vaped					
Earlier today	432 (42.3)	44 (38.9)	89 (33.6)	132 (47.0)	167 (46.1)
Not today, but sometime during the past 7 days	231 (22.6)	22 (19.5)	68 (25.7)	57 (20.3)	84 (23.2)
Not during the past 7 days, but sometime during the past 30 days	98 (9.6)	17 (15.0)	29 (10.9)	28 (10.0)	24 (6.7)
Not during the past 30 days, but sometime during the past 6 months	40 (3.9)	7 (6.2)	13 (4.9)	12 (4.3)	8 (2.2)
Not during the past 6 months, but sometime during the past year	101 (9.9)	8 (7.1)	33 (12.4)	26 (9.2)	34 (9.4)
1 to 4 years ago	65 (6.4)	8 (7.1)	19 (7.2)	10 (3.5)	28 (7.7)
5 or more years ago	47 (4.6)	6 (5.3)	14 (5.3)	14 (5.0)	13 (3.6)
Did not answer	7 (0.7)	1 (0.9)	0 (0.0)	2 (0.7)	4 (1.1)
How long does it take to finish 1 non-nicotine vape?					
Less than a day	80 (7.8)	6 (5.3)	17 (6.4)	22 (7.8)	35 (9.7)
1-2 days	139 (13.6)	7 (6.2)	28 (10.6)	34 (12.2)	70 (19.3)
3-5 days	274 (26.8)	24 (21.3)	73 (27.5)	71 (25.3)	106 (29.3)

© 2022 Gaiha SM et al. *JAMA Network Open.*

A week (7 days)	257 (25.2)	25 (22.1)	70 (26.4)	81 (28.8)	81 (22.4)
Two weeks	105 (10.3)	12 (10.6)	27 (10.2)	34 (12.1)	32 (8.8)
One month	79 (7.8)	14 (12.4)	26 (9.8)	20 (7.1)	19 (5.3)
Don't know	54 (5.3)	18 (15.9)	15 (5.7)	13 (4.6)	8 (2.2)
Did not answer	33 (3.2)	7 (6.2)	9 (3.4)	6 (2.1)	11 (3.0)
How likely is it that over the next 6 months you will use a non-nicotine vape again? (anything except very unlikely)	835 (81.8)	81 (71.7)	204 (76.9)	229 (81.5)	321 (88.7)

[a] Responses were missing for up to 3.2% of participants who were asked questions about past 30-day use and reported as "Did not answer."

© 2022 Gaiha SM et al. *JAMA Network Open.*

eTable 2. Brands Used Among Ever Users (n=1590) and Past 30-Day Users (n=1021) of Nonnicotine e-Cigarettes, by Age, n (%)

	Ever-users[a]					Past 30-day users				
	Total[b] (N = 1590)	13-17 years (N = 227)	18-20 years (N = 497)	21-24 years (N = 399)	25-40 years (N = 467)	Total (N = 1021)	13-17 years (N = 113)	18-20 years (N = 265)	21-24 years (N = 281)	25-40 years (N = 362)
Cloudy	790 (49.7)	88 (38.8)	243 (48.9)	220 (55.1)	239 (51.2)	233 (22.8)	23 (20.4)	50 (18.9)	78 (27.8)	82 (22.7)
Vitaminvape	758 (47.7)	71 (31.3)	194 (39.0)	224 (56.1)	269 (57.6)	242 (23.7)	13 (11.5)	50 (18.9)	77 (27.4)	102 (28.2)
Breathe	714 (44.9)	66 (29.1)	210 (42.3)	202 (50.6)	236 (50.5)	214 (21.0)	14 (12.4)	56 (21.1)_	56 (19.9)	88 (24.3)
LUVV	713 (44.8)	63 (27.8)	198 (39.8)	198 (49.6)	254 (54.4)	219 (21.4)	14 (12.4)	44 (16.6)	63 (22.4)	98 (27.1)
HealthVape	709 (44.6)	67 (29.5)	183 (36.8)	200 (50.1)	259 (55.5)	220 (21.5)	18 (15.9)	46 (17.4)	70 (24.9)	86 (23.8)
Monq	701 (44.1)	59 (26.0)	206 (41.4)	204 (51.1)	232 (49.7)	215 (21.1)	15 (13.3)	48 (18.1)	64 (22.8)	88 (24.3)
Nutrovape	688 (43.3)	56 (24.7)	194 (39.0)	189 (47.4)	249 (53.3)	213 (20.9)	16 (14.2)	47 (17.7)	61 (21.7)	89 (24.6)
Inhale Health	687 (43.2)	60 (26.4)	202 (40.6)	184 (46.1)	241 (51.6)	213 (20.9)	12 (10.6)	53 (20.0)	61 (21.7)	87 (24.0)
MOXĒ	687 (43.2)	59 (26.0)	193 (38.8)	192 (48.1)	243 (52.0)	205 (20.1)	11 (9.7)	43 (16.2)	59 (21.0)	92 (25.4)
Ripple+	686 (43.1)	61 (26.9)	203 (40.8)	191 (47.9)	231 (49.5)	220 (21.5)	18 (15.9)	47 (17.7)	63 (22.4)	92 (25.4)
Fum	672 (42.3)	61 (26.9)	191 (38.4)	185 (46.4)	235 (50.3)	222 (21.7)	16 (14.2)	50 (18.9)	63 (22.4)	93 (25.7)
Teatime	669 (42.1)	55 (24.2)	179 (36.0)	197 (49.4)	238 (51.0)	213 (20.9)	14 (12.4)	50 (18.9)	63 (22.4)	86 (23.8)

© 2022 Gaiha SM et al. *JAMA Network Open.*

Vitastik	668 (42.0)	54 (23.8)	187 (37.6)	196 (49.1)	231 (49.5)	216 (21.2)	9 (8.0)	53 (20.0)	65 (23.1)	89 (24.6)
Eagle Energy	666 (41.9)	53 (23.3)	191(38.4)	186 (46.6)	236 (50.5)	209 (20.5)	17 (15.0)	47 (17.7)	55 (19.6)	90 (24.9)
Corked	645 (40.6)	46 (20.3)	188 (37.8)	184 (46.1)	227 (48.6)	205 (20.1)	15 (13.3)	49 (18.5)	55 (19.6)	86 (23.8)

[a]Ever use includes past 30-day use; [b] Responses were missing for up to 5.4% of total ever users who did not identify brands used. Proportions were calculated with missing responses included in the denominators.

© 2022 Gaiha SM et al. *JAMA Network Open.*

eTable 3. Reported Ingredients in Nonnicotine e-Cigarettes, by Brand and Product

Brand	Product	Ingredient List
VitaminVape	VitaminVape	Vegetable Glycerin USP, Deionized Water, Organic Fruit Flavor Extract (Water, Organic Ethyl Alcohol, and Natural Flavors), Vitamin B12 (Cyanocobalamin)
Breathe	Vitamin B12 Diffuser	Vegetable Glycerin USP, Deionized Water, Organic Fruit Flavor Extract (Water, Organic Ethyl Alcohol, and Natural Flavors), Vitamin B12 (Methylcobalamin)
Cloudy	Calmies	Magnesium Sulfate, Vitamin B6, L-Theanine, DHA (Algal Oil), CoQ10, Vitamin B12, Folic Acid (as 5-MTHF), Ashwagandha, Valerian, Chamomile, Lemon Balm, Lavender, Rhodiola Rosea Root, Pectin
	Calming Cream	Aloe Leaf Juice, Shea Butter, Emulsifying Wax, Jojoba Oil, Lavender Distillate, Mango Butter, Evening Primrose Oil, Witch Hazel Distillate, Alcohol, Willow Bark Extract, Vitamin E, Neem Seed Oil, Rosemary Leaf Extract, Sunflower Seed Oil, Benzoin Resin, Bulgarian Lavender Essential Oil, Natural Fragrance, Stearic Acid, Citric Acid, Xanthan Gum, Tetrasodium Glutamate Diacetate, Phenoxyethanol
	Dream Mist	Aloe Leaf Juice, Polysorbate 20, Polysorbate 80, Lavender Distillate, Witch Hazel Distillate, Benzoin Resin, Bulgarian Lavender Essential Oil, Lavandin Grosso Essential Oil, Ylang Ylang Essential Oil, Clary Sage Essential Oil, Eugenol, Bitter Almond Essential Oil, Patchouli Essential Oil, Alcohol, Tetrasodium Glutamate Diacetate, Phenoxyethanol
	Melatonin Diffuser	Melatonin, Lavender Extract, L-Theanine, Chamomile Extract, Grape Extract, Propylene Glycol, Vegetable Glycerin
	Mellows	Magnesium Sulfate, Vitamin B6, L-Theanine, Melatonin, Ashwagandha, Valerian, Chamomile, Lemon Balm, Lavender Extract, GABA, Pecti
HealthVape	Boost	Vitamin B12, Vitamin B6, L-Theanine, L-Lysine, Taurine, Wild Berry and Peppermint flavors

© 2022 Gaiha SM et al. *JAMA Network Open.*

	Chill	Chamomile, Valerian Root, L-Theanine, Passionflower, Lavender, Orange, Geranium and Cassia flavors
	Energy	Caffeine, Vitamin B12, L-Theanine, L-Carvone, Lemon, Cassia and Mandarin Orange flavors
	Restore	Collagen, Glutathione, L-Carnitine, b-Ionone, Geranium, Lavender, Cassia and Mandarin Orange flavors
	Soothe	Melatonin, Passionflower, Chamomile, Valerian Root, L-Theanine, Peppermint, Mandarin Orange and Cassia flavors
	Vital	Co-Enzyme Q10, Vitamin C, Vitamin D3, Vitamin B12, Vitamin A, Lime and Zesty Mint flavors
LUVV Air Inhalers	Calm	Melatonin, Passionflower, Chamomile, Valerian Root, L-Theanine, Peppermint and Cassia flavors
	Glow	Collagen, L-Carnitine, Geranium, L-Carvone, b-Ionone, Peppermint and Cassia flavors
	Rise	Vitamin B12, Vitamin B6, L-Theanine, L-Lysine, Vegan-Friendly Taurine, Wild Berry and Mint extract
	Vita	Co-Enzyme Q10, Vitamin C, Vitamin D3, Vitamin B12, Vitamin A, Mint, Lime, Citrus flavors
	Wake	Caffeine, Vitamin B12, L-Theanine, L-Carvone, Mandarin orange, cassia and Lemon flavors
	Zen	Chamomile, Valerian Root, Passionflower, L-Theanine, Flower Plant Daytime Photograph White Light Botany Green Nature Petal, Lavender flowers, Lavender, Geranium and Cassia flavors
MONQ	Bright	Lemon, Lime and Clove
	Cheer	Frankincense, Myrrh, Palo Santo
	Cozy	Cardamom, Cinnamon Leaf, Vanilla
	Delight	Spicy Sassafras and Anise, Vanilla, Wintergreen (original)
	Forest	Fir, Frankincense, and Pine
	Fresh	Ginger, Lemon, Peppermint
	Happy	Fennel, Thyme, Linalool, Vanilla, Caraway Seed, Clary Sage, Green Mandarin, Lavender, Mandarin Petitgrain, Marjoram & Parsley Seed
	Joy	Orange and Pink peppercorn
	Lively	Coriander, Orange, and Vanilla
	Love	Cacao, Peppermint, Vanilla
	Mountain	Juniper Leaf, Peppermint, and Scotch Pine

© 2022 Gaiha SM et al. *JAMA Network Open.*

	Ocean	Eucalyptus, Lime, Tangerine, Atlas Cedarwood, Bergamot, Cypress Leaf, Douglas Fir, Frankincense, Geranium, Juniper Leaf, Lavender, Lemon, Lemongrass, Litsea, Manuka, Neroli, Peppermint, Scotch Pine & Ylang-Ylang
	Peace	Frankincense, Rosemary, Yellow Mandarin
	Pumpkin Spice	Allspice, Cinnamon Leaf, Nutmeg
	Relieve	Relieve: Ginger, Helichrysum, Spikenard, Bergamot, Black Pepper, Copaiba, Frankincense, Geranium, Jasmine, Lavender, Nerolina, Ravensara & Roman Chamomile
	Sexy	Cinnamon Leaf, Jasmine, Patchouli
	Sleepy	Bergamot, Chamomile, Lavender, Lemongrass, Marjoram, Neroli & Sandalwood
	Zen	Frankincense, Sweet Orange, Ylang-Ylang, Black Pepper, Clary Sage, Dill Seed, Eucalyptus, Lemon, Rosemary, Sage, Spearmint, Tea Tree & Glycerin
Inhale Health	Anti-cigarette	Menthol, Vegetable glycerin
	Caffeine	Caffeine, Vegetable glycerin
	Melatonin	Melatonin, Vegetable base
	Vitamin B12	Vitamin B12, Vegetable base
Teatime	Various flavors	Flavorings (Cherry, Vanilla, Blueberry, Peppermint, Citrus, Mint, Peach, Mango, Apple spice, Raspberry, Earl Grey, Mixed berry, Strawberry, Hibiscus, Green tea)
EagleEnergy	Lychee Ice/ Guarana Berry	Caffeine, Vitamin B12, Panax Ginseng Extract, Natural and Artificial Flavors (Lychee, Mint, Guarana)
MOXĒ	Blaze	Cinnamon Bark (Cinnamomum zeylanicum), Frankincense (Boswellia serrata), Nutmeg (Myristica fragrans), Cardamom (Elettaria cardamomum), Clove (Syzgium aromaticum), Black Pepper (Piper nigrum), Capsicum (Capsicum annuum)
	Breathe	Peppermint (Mentha arvensis), Eucalyptus (Eucalyptus globulus), Cajeput (Melaleuca cajeputi), Tea Tree (Melaleuca alternifolia), Rosemary (Rosmarinus officinalis), Lavender (Lavandula angustifolia), Pink Himalayan Salt

© 2022 Gaiha SM et al. *JAMA Network Open.*

	Dream	Lavender (Lavandula angustifolia), Lemongrass (Cymbopogan citratus), Grapefruit (Citrus x paradisi)
	Energy	Spearmint (Mentha spicata), Grapefruit (Citrus paridisi), Bergamot (Citrus bergamia), Sandalwood (Santalum alb
	Frost	Peppermint (Mentha balsamea), Eucalyptus (Eucalyptus globulus), Camphor (Cinnamomum camphora), Wintergreen (Gaultheria procumbens), Tea Tree (Melaleuca alternifolia)
	Happy	Fennel (Foeniculum vulgare), Thyme (Thymus vulgaris), Cinnamon (Cinnamomum verum), Sage (Salvia officinalis)
	Peace	Bergamot (Citrus bergamia), Mandarin (Citrus reticulata), Geranium (Pelargonium graveolens), Lime (Citrus x aurantiifolia)
	Love	Rose (Rosa damascena), Jasmine (Jasminum sambac), Sandalwood (Santalum album), Bergamot (Citrus bergamia), Lemon (Citrus limonum), Lime (Citrus latifolia)
Corked	Alive	Essential oils (Caraway, Cinnamon leaf, Clary sage, Dill seed, Ginger, Lemon, Lime peel, Mandarin, Marjoram, Petitgrain, Rosemary, Tea tree, Thyme, Ylang ylang), Vanilla oleoresin absolute, Vegetable glycerin, Vitamin B12
	Ecstacy	Essential oils (Black pepper, Caraway, Cinnamon leaf, Copaiba, Frankincense, Geranium, Ginger, Hyssop, Lemon, mandarin, orange), vanilla oleoresin absolute, vegetable glycerin, vitamin b12
	Meditate	Essential oils (Bergamot, Bitter orange, Clary sage, Copaiba, Dill seed, Eucalyptus, Frankincense, Hyssop, Lavender, Lemongrass, Marjoram, Orange, Parsley seed, Peppermint, Sage, Sweet marjoram, Turmeric, Valerian root, Ylang ylang), Vegetable glycerin, Vitamin B12
	Refresh	Essential oils (Bergamot, Bitter orange, Clary sage, Copaiba, Dill seed, Eucalyptus, Frankincense, Hyssop, Lavender, Lemongrass, Marjoram, Orange, Parsley seed, Peppermint, Sage, Sweet Marjoram,

© 2022 Gaiha SM et al. *JAMA Network Open.*

		Turmeric, Valerian root, Ylang ylang) vegetable glycerin, Vitamin B12
	Trance	Essential oils (Bergamot, Bitter orange, Copaiba, Dill seed, Hyssop, Lavender, Lemongrass, Mandarin, Peppermint, Petitgrain, Sweet marjoram, Thyme, Valerian root, Ylang ylang), Vegetable glycerin, Vitamin B12
Ripple+	Boost	Green tea extract, Maca root extract, Natural flavorings, Vegetable glycerin, Propylene glycol
	Dream	Valerian extract, Jujube seed extract, Natural flavorings, Vegetable glycerin, Propylene glycol
	Focus	Bacopa extract, Guarana extract, Natural flavorings, Vegetable glycerin, Propylene glycol
	Power	Ginseng extract, Gingko biloba extract, Natural flavorings, Vegetable glycerin, Propylene glycol
	Relax	Chamomile extract, Lemon balm extract, Natural flavorings, Vegetable glycerin, Propylene glycol
	Revive	Ashwagandha extract, Rhodiola extract, Natural flavorings, Vegetable glycerin, Propylene glycol
Füm	Bubbly Lime	Essential oils (Cedarwood, Cinnamon, Lemon, Lime, Spearmint)
	Conquer	Essential oils (Black Pepper, Clove, Peppermint, Eucalyptus Globulus, Cinnamon, Lime, Cedarwood, Lavender, Copaifera reticula balsam, Ocotea quixos leaf), Vanilla planifolia extract
	Cozy Chai	Essential oils (Cinnamon, Nutmeg, Clove, Orange, Cedarwood)
	Invigorate	Essential oils (Davana, Cedarwood, Copaiba, Eucalyptus globulus, Eucalyptus radiata, Grapefruit, Lime, Lavender, Ocotea, Peppermint), vanilla planifolia extract
	Lemon berry bliss	Essential oils (Eucalyptus radiata, Lavender, Lemon, Lemongrass, Peppermint, Spearmint, Ylang-ylang)
	Reassurance	Essential oil (Lavender, Cinnamon, Orange, Lime, Cedarwood, Lavender, Vanilla planifolia fruit extract, Copaifera reticula balsam, Ocotea quixos leaf)

© 2022 Gaiha SM et al. *JAMA Network Open.*

	Shield	Essential oils (Clove, Peppermint, Eucalyptus globulus, Lemon, Orange, Cinnamon, Tangerine)
	White cranberry	Essential oils(Black Pepper, Black Spruce, Cinnamon, Davana, Grapefruit, Juniper, Orange)
Vitastik	Amore	Rose geranium oil, Vitamin C, Vitamin B2, Vitamin B6, Vitamin B12
	Beauty	Spearmint, Watermelon, Rose oils, Vitamin C, Vitamin B2, Vitamin B6, Vitamin B12
	Breathe	Eucalyptus, Menthol, Basil, Mint, Myrrh essential oils, Ginseng, Vitamin C, Vitamin B2, Vitamin B6, Vitamin B12
	Calm	Crushed vanilla, Rose, Ginger, Green-tea, Vitamin C, Vitamin B2, Vitamin B6, Vitamin B12, Valerian Root
	Energy	Ginseng, Grapefruit, Lemon, Orange, Vitamin B12, Vitamin B2, Vitamin B6, Vitamin C, CoQ10
	Flora	Hibiscus flowers, Vanilla, Mint, Ylang-ylang essential oils, Vitamin B12, Vitamin B2, Vitamin B6, Vitamin C
	Focus	Coffee oils, Ginkgo Biloba, Sandalwood, Vetiver, Ginseng, Vitamin B-Complex, Vitamin C, CoQ10, Vitamin B12
	Handsome	Coconut, Damiana, Horny goat weed, Vitamin B12, Vitamin B2, Vitamin B6, Vitamin C
	Relax	Blackberry, Boysenberry, Blueberry, Grape water, Lavender, Rose, Ylang-ylang essential oil, Vitamin C, Vitamin B2, Vitamin B6, Vitamin B12
	Silver Bullet	Menthol, Ginseng, Vitamin B12, Vitamin B2, Vitamin B6, Vitamin C
	Sleep	Melatonin, Lavender, Honey, Vitamin B12, Vitamin B2, Vitamin B6, Vitamin C
	Wellness Tonic	Ginger, Raw honey, Chamomile lemon tea, Reishi mushroom extract, Vitamin B12, Vitamin B2, Vitamin B6, Vitamin C

Product ingredients are collected from a brand's published websites. This table does not represent a comprehensive list of ingredients found in each vape product; products may contain other unlisted ingredients. Ingredients in this table are not listed in any particular order.

© 2022 Gaiha SM et al. *JAMA Network Open.*
